# Exosomes in the Pathogenesis and Treatment of Multiple Myeloma in the Context of the Bone Marrow Microenvironment

**DOI:** 10.3389/fonc.2020.608815

**Published:** 2020-11-05

**Authors:** Tianzeng Chen, Maria Moscvin, Giada Bianchi

**Affiliations:** ^1^Division of Hematology, Department of Medicine, Brigham and Women’s Hospital, Boston, MA, United States; ^2^Harvard Medical School, Boston, MA, United States

**Keywords:** exosome, multiple myeloma (MM), bone marrow, microenvironment, pathogenesis, emerging roles

## Abstract

Multiple myeloma (MM), the second most common hematological malignancy, is an incurable cancer of plasma cells. MM cells diffusely involves the bone marrow (BM) and establish a close interaction with the BM niche that in turn supports MM survival, proliferation, dissemination and drug resistance. In spite of remarkable progress in understanding MM biology and developing drugs targeting MM in the context of the BM niche, acquisition of multi-class drug resistance is almost universally inevitable. Exosomes are small, secreted vesicles that have been shown to mediate bidirectional transfer of proteins, lipids, and nucleic acids between BM microenvironment and MM, supporting MM pathogenesis by promoting angiogenesis, osteolysis, and drug resistance. Exosome content has been shown to differ between MM patients and healthy donors and could potentially serve as both cancer biomarker and target for novel therapies. Furthermore, the natural nanostructure and modifiable surface properties of exosomes make them good candidates for drug delivery or novel immunomodulatory therapy. In this review we will discuss the current knowledge regarding exosome’s role in MM pathogenesis and its potential role as a novel biomarker and therapeutic tool in MM.

## Introduction

Multiple myeloma is the second most common hematological malignancy in the Western world after non-Hodgkin lymphoma, accounting for approximately 13% of all hematological cancers (1). About 32,270 new cases of MM and 12,830 MM-related deaths are expected in 2020 (2). Although autologous stem-cell transplantation and agents targeting both MM and the BM niche, such as immunomodulatory drugs (IMiDs), proteasome inhibitors, and monoclonal antibodies, have profoundly extended the overall survival of MM patients, the disease remains incurable (3). Multiclass relapsed/refractory MM patients face a dismal prognosis with limited therapeutic options, and thus there is an urgent need to understand better the biology of MM and explore new therapeutic approaches (4).

MM is characterized by end organ damage caused by monoclonal expansion of malignant plasma cells within the BM, associated with an excess of monoclonal protein in the blood or urine (5). Recent data show that MM consistently progresses from a precursor state of monoclonal gammopathy of undetermined significance (MGUS) or smoldering multiple myeloma (SMM) (6, 7). The facts that the primary oncogenic mutations observed in MM patients are already present in MGUS or SMM and that genomic landscape appears remarkably similar across the plasma cell disorder spectrum, suggest that the BM microenvironment may play a crucial role in disease progression from an asymptomatic state to malignant neoplasms (8, 9).

It has been widely demonstrated that the BM microenvironment promotes tumor growth, angiogenesis, and osteolysis (9). Among various interactions within the bone marrow, recent studies reveal that exosomes are important cross-talking mediators during tumor growth and progression (10). Exosomes are small (30-100nm diameter) membrane vesicles generated in multivesicular endosomes (MVEs) and released upon the fusion of MVEs with cell membrane (11, 12). These nano vesicles are secreted by most cell types under both physiological and pathological conditions, mediating local and systemic cell-to-cell communication through selective transfer of mRNA, non-coding RNA (ncRNA), proteins, and lipids (13, 14). There is a growing interest in understanding how exosomes contribute to MM pathogenesis and if they could be use as a therapeutic vehicle in MM treatment.

## Exosome Biogenesis

Exosome biogenesis starts in the endosomal system as endosomes accumulate intraluminal vesicles and mature into multivescular endosomes (MVEs or MVBs) (15, 16). During the process, cargo macromolecules including lipids, proteins, and nucleic acids are clustered and recruited *via* ESCRT (endosomal sorting complex required for transport)–dependent or ESCRT-independent mechanisms (12, 15). Once matured, MVEs that are not destined for degradation are transported along microtubules and docked to the plasma membrane, after which exosomes are released upon the fusion of MVEs and the plasma membrane (12, 16). When exosomes reach the recipient cells, they exert their effects by binding to the cell surface and triggering downstream intracellular signaling; by fusing directly with the plasma membrane to deliver cargos; or by being internalized through pathways such as endocytosis and phagocytosis (12).

**Figure 1:**
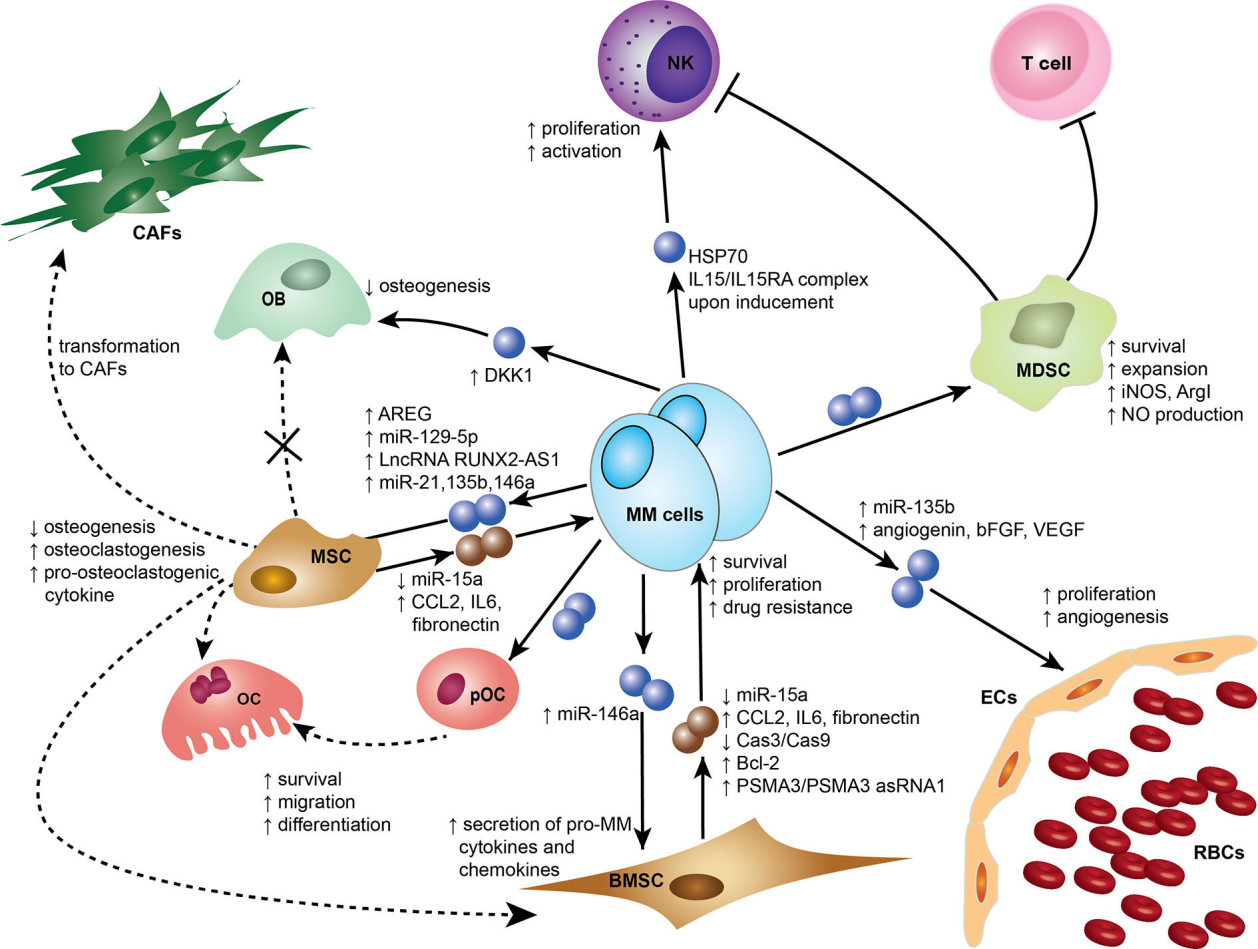
Exosomes mediate cross-cell communication in the multiple myeloma (MM) bone marrow microenvironment (BMM). Tumor-derived exosomes remodel stromal cells, affect osteogenesis, induce angiogenesis, and help create an immunosuppressive microenvironment. Small dark blue spheres represent MM-derived exosomes and brown spheres represent MSC/BMSC-derived exosomes contribute to MM survival, proliferation, and drug resistance. Dotted arrows indicate differentiation, whereas solid arrows indicate effects on a target cell. Cells and associated effects are shown. BMSC, Bone marrow stromal cell; CAF, Cancer-associated fibroblast; ECs, Endothelial cells; MSC, Mesenchymal stem cell; MDSC, myeloid-derived suppressor cells; NK, Natural killer cell; OB, Osteoblast; OC, Osteoclast; pOC, Pre-osteoclast cell; RBCs, Red blood cells. AREG, amphiregulin; ArgI arginase 1; bFGF, basic fibroblast growth factor; Bcl-2, B-cell lymphoma 2; Cas3, Caspase-3; Cas9, Caspase-9; CCL2, chemokine C-C motif ligand 2; HSP70, Heat Shock Protein 70; IL6, Interleukin 6; IL15 Interleukin 15; IL15RA IL15 Receptor Subunit Alpha; iNOS, Inducible nitric oxide synthase; NO, Nitric oxide; PSMA3, Proteasome subunit alpha type-3; PSMA3 asRNA1, PSMA3 antisense RNA1; VEGF, Vascular endothelial growth factor; LncRNA RUNX2-AS1, Long non-coding RNA Runt-related transcription factor 2 antisense RNA1.

Exosomes were initially thought to be means for cells to eliminate unwanted materials, but they are now considered more as biological active entities that play a role in intercellular communication and contribute to many physiological and pathological functions (16–19). In recent reports, exosomes have been shown to be an important element mediating cell recruitment, immunosuppressive effects, and horizontal transfer of genetic information either locally or systemically to ensure continuous crosstalk between the tumor and its microenvironment (18, 20). Emerging evidence supports that MM-derived exosomes (MM-EXs) reprogram recipient cell functions in the BM to modulate and mold a pro-tumor environment capable of supporting disease progression (21). MM-EXs affect the function of several components of the BM milieu, including natural killer (NK) cells, myeloid-derived suppressor cell (MDSC), mesenchymal stem cells (MSC), endothelial cells, osteoblast (OB), and osteoclast (OC) (**Figure 1**). Exosome signaling is bidirectional and bone marrow stromal cells (BMSCs)-derived exosomes (BMSC-EXs) have been shown to induce MM growth, survival, and drug resistance (22).

## Exosomes Role in MM Pathogenesis

Roccaro et al. demonstrated that BMSC-EXs obtained from MM patients promoted tumor growth while BMSC-EXs from healthy donors inhibited MM cell proliferation (23, 24). Exosome profiling showed MM-BMSC-EXs expressed a lower level of tumor-suppressive factor miRNA-15a, and a higher level of chemokine C-C motif ligand (CCL) 2, interleukin (IL) 6, and fibronectin, which play a crucial role in MM pathogenesis and tumor progression (24). A distinct study similarly confirmed the difference in exosomal content between normal and MM in the 5T33 murine model (25). BMSC selectively transferred certain proteins into MM cells that induced p38, p53, c-Jun N-terminal kinase (JNK), and Akt pathways to promote MM cell survival (25). Interestingly, Wang et al. also reported that exosomes obtained from both normal donor and MM patient BMSCs induced drug resistance of human MM cells. They proposed that BMSC-EXs-mediated upregulation of anti-apoptotic B-cell lymphoma (Bcl)-2 and downregulation of apoptotic Caspase 9 and Caspase 3 in MM cells inhibited spontaneous and bortezomib-induced apoptosis (25). Recently, a study showed that BMSC-derived exosomes from PI-resistant MM patients transferred PSMA3 and PSMA3 Antisense RNA1 to MM cells, causing increased proteasome activity and thus mediating PI resistance (26).

Rather than a one-way order, the mutual communication between MM and BMSC cells *via* exosomes enables a feedback loop in the BM microenvironment to support MM progression. Our group showed that co-culture of MM induced HDAC3 expression in BMSC cells, while HDAC3 knock down in BMSC lead to quantitative and qualitative changes in secreted exosomes that contributed to MM cell growth arrest (27). Moreover, De Veirman et al. examined the miRNA changes in human MSC after culture with conditioned medium of MM cells and found 19 dysregulated miRNAs, including upregulated miR-146a. They further demonstrated that exosomes transferred miR-146a from MM cells into MSC. In return, the overexpression of miR-146a in MSC increased the secretion of cytokines and chemokines including C-X-C motif chemokine ligand (CXCL) 1, CXCL10, IL6, IL8, CCL2, and CCL5, resulting in the enhancement of MM cell viability and migration (28).

Uptake of BMSC-derived exosomes by MDSCs in MM patients results in accelerated tumor growth and generation of an immunosuppressive BM milieu. An *in vitro* study showed that BMSC-derived exosomes induced the survival and expansion of MDSCs through activating signal transducer and activator of transcription (STAT) 3 and STAT1 pathways and increasing the anti-apoptotic proteins B-cell lymphoma-extra large (Bcl-xL) and induced myeloid leukemia cell differentiation protein Mcl-1 (29). The same group also showed that exosomes from BMSCs further activated MDSCs *in vivo* to increase their nitric oxide production, which contributed to the inhibition of T cells (29, 30). They later showed that MM-EXs also activated STAT3 in MDSCs to express high levels of both arginase 1 and inducible nitric oxide synthase, which enhanced T-cell suppression (31).

## Exosome Impact on BM Microenvironment Remodeling

Osteolysis is a common characteristic of MM resulting from a disrupted equilibrium between OBs and OCs, which are responsible for new bone apposition and bone resorption, respectively to guarantee adequate bone mass (32, 33). Exosomes in the BM have been shown to contribute to this pro-OC microenvironment, resulting in impaired bone formation and MM-related bone disease. Emerging evidence suggests that exosomal ncRNAs play an important role in this regard (34, 35).

Raimondi et al. were the first to show that exosomes derived from MM cells and MM patient’s sera directly influenced OCs differentiation and function. MM-EXs not only supported migration of pre-osteoclast cells (pOCs) through the increasing of C-X-C chemokine receptor type 4 expressions, but also induced their differentiation into multinuclear OCs with specific OCs markers such as cathepsin K, matrix metalloproteinases 9 (MMP9), and tartrate-resistant acid phosphatase (TRAP). Besides promoting their bone resorptive activity, MM-EXs suppressed apoptosis of pOCs and enhanced their survival by activating the Akt pathway (36). Recent studies confirmed those observations and further explored the effective contents in MM-EXs and their mechanisms to exert functions on MSCs. LncRNA RUNX2 antisense RNA 1 (RUNX2-AS1), amphiregulin, and miR-129-5p were identified to be specifically enriched in MM-EXs. Upon internalization of the exosomes by MSC, these molecules reduced RUNX2 splicing efficiency, activated the epidermal EGFR pathway, and downregulated the expression of the transcription factor Sp1, respectively (37–39). Each pathway has been demonstrated to decrease the osteogenic potential of MSCs, increase osteoclastogenesis, and contribute to osteoblast deficiency. A new study also identified UPR (unfolded protein response)-related signaling molecules in MM-EXs that were proposed to induce osteoclastogenesis through activation of the XBP1/IRE1α axis (40). Additionally, MM-EXs promoted secretion of pro-osteoclastogenic cytokine IL8 and IL6 *via* APE1/NF-κB pathway and suppressed osteoblastic differentiation proteins Runt-related transcription factor 2 (Runx2), Osterix and osteocalcin (38, 39, 41). Exosomes derived from 5TGM1, murine MM cells also demonstrated the ability to block osteoblast differentiation and functionality *in vitro*. Faict and colleagues suggested that the transfer of dickkopf WNT signaling pathway inhibitor 1 (DKK1) triggered inactivation of the Wnt signaling pathway that lead to a reduction in Runx2, Osterix, and Collagen 1A1 in osteoblasts (42).

Several studies indicated that soluble factors from MM cells stimulated the overexpression of miR-135b in MSCs, which was associated with the negative regulation of MSCs osteogenesis and their impaired osteogenic differentiation ability in MM patients (43). Umezu et al. later showed that exosomes from chronic hypoxia-resistant MM (HR-MM) cells, which mimicked tumor cells from the BM, were highly enriched in miR-135b (44). They provided new evidence that exosomal miR-135b directly suppressed factor inhibiting HIF-1 (FIH-1) to accelerate hypoxia-inducible factor (HIF)-1 transcriptional activity in endothelial cells and attributed to hypoxia-driven accelerated tube formation (44, 45). As BM is highly vascularized and naturally hypoxic and the MM-infiltrated BM even more hypoxic due to the massive proliferation of MM cells, exosomes target it primarily to increase angiogenesis (46, 47). Using the same HR-MM model both *in vitro* and *in vivo*, Umezu demonstrated that miR-340 from healthy BMSC exosomes inhibited angiogenesis *via* the hepatocyte growth factor/c-MET (HGF/c-MET) signaling pathway in endothelial cells. However, BMSC exosomes from older donors with senescent profiles were less effective in reducing angiogenesis (48). In murine models, Wang et al. confirmed a strong pro-angiogenic effect of MM-EXs and identified multiple angiogenic factors as cargo proteins, including angiogenin, basic fibroblast growth factor (bFGF), and vascular endothelial growth factor (VEGF) (31, 49). They further demonstrated that MM-EXs enhanced phosphorylation of Stat3, JNKs, and p53 in endothelial cells and directly facilitate their growth (31).

## Diagnostic and Prognostic Role of Exosomes

Accurate diagnosis and prognosis with a close monitor of disease progression are essential in designing appropriate therapy for patients. There are significant research interests in identifying biomarkers and other non-invasive approaches for diagnosis and disease classification to facilitate patient follow up and care. As previously shown, exosomes are actively secreted by cells and can be isolated from the peripheral blood, making them suitable candidates as biomarkers (50). Proteomic characterization of exosomes secreted by different MM cell lines revealed that they contained a common pattern of proteins and thus could potentially represent an important tool to detect low burden disease (51). Exosomal lncRNA profiling distinguished MM and MGUS patients from healthy donors (14, 52). MicroRNAs are also important components of exosomes, delivering tumor-promoting messages and impacting signaling and protein expression in target cells (53). A study of circulating exosomal miRNAs isolated from the serum of 156 patients identified 22 miRNAs expressed at a significantly lower level in MM patients compared to healthy individuals. Among those, let-7b and miR-18a were significantly associated with both progression-free survival and overall survival. Patients characterized by lower exosomal let-7b and miR-18a levels were more likely to present with high stage in the International Staging System and have a poor outcome (54). Another study found that miR-129-5p, which targeted OBs differentiation markers, was enriched in exosomes from MM patients compared to those from SMM patients, suggesting exosomes may be a useful marker of disease progression (39). Higher expression of exosomal miR-214 detected in osteoporotic patients also suggested exosome’s potential as a biomarker for MM bone diseases (34, 35). Further validation in other independent MM patient cohorts will explore the potential of circulating exosomal miRNAs to improve the prognostic and risk stratification.

Tools to predict drug resistance are becoming increasingly important in the era of personalized medicine. Zhang et al. focused on the predictive value of exosomal miRNA for primary or acquired drug resistance in MM patients. They analyzed 204 patients data and discovered that exosomal miR-16-5p, miR-15a-5p and vmiR-20a-5p, miR-17-5p were downregulated in patients resistant to bortezomib (55). Another study identified circulating exosomal PSMA3 and PSMA3-AS1 as clinically relevant biomarkers correlated with PI resistance. Newly diagnosed, MM patients with a low exosomal expression of PSMA3 and PSMA3-AS1 were sensitive to bortezomib, whereas patients with a high expression responded poorly (26).

Allogeneic hematopoietic stem cell transplantation (HSCT) is a treatment strategy that can be carefully considered in young patients with aggressive MM as a tool to achieve long-term disease remission. However, transplant-related complications, primarily acute and chronic graft-vs-host disease (GVHD) are a substantial cause of morbidity and mortality (56). Lia et al. conducted an exploratory study of 41 MM patients undergoing allogeneic HSCT to investigate exosomal surface antigens as potential predicative biomarkers for acute GVHD. CD146 correlated with a 60% increased risk of developing GVHD, whereas CD31 and CD140-α with a 40% and 60%, respectively, reduced risk (57).

## Exosome-Related Targets and Therapies

Exosomes are important message carriers that contribute to generate a tumor permissive microenvironment in the BM. A number of studies have investigated the therapeutic potential of targeting exosome secretion in MM. In murine MM models, sphingomyelinase inhibitor GW4869 blocked exosome secretion, preventing exosome-mediated bone lesions and increasing cortical bone volume. Importantly, GW4869 also strongly synergized with bortezomib in mediating anti-myeloma activity, suggesting that perturbation of exosomes can directly affect MM survival and proliferation (37, 42). However, ceramide C6 (C6-cer), an exogenous ceramide supplement, dose-dependently increased MM exosome secretion but inhibited cell proliferation and induced apoptosis (58). Interestingly, after C6-cer treatment, Cheng et al. detected decreased levels of tumor suppressive miRs including miR 202, miR 16, miR 29b, and miR 15a in MM cells and increased levels of these miRs in exosomes. While discrepancy of alterations in intracellular miRs and MM proliferation and apoptosis upon C6-cer treatment still requires further investigation, they demonstrated that those MM-EXs with elevated tumor-suppressive miRs exhibited paracrine effects on recipient MM cells that suppress tumor growth (58).

Apart from targeting exosome secretion, disrupting interactions between exosomes and recipient cells to prevent exosome uptake or content loading also has therapeutic value. Purushothaman et al. discovered that heparan sulfate plays a dual role in exosome-cell interaction, capturing fibronectin on exosomes and acting as a receptor for fibronectin on target cells. Fibronectin-mediated binding of exosomes to target cells can trigger signaling pathways like p38 and pERK and downstream expression of DKK-1 and MMP-9, two molecules with well-known roles in MM progression (59). They further showed that removal of heparan sulfate with bacterial heparitinase or using antibody specific for the Hep-II heparin-binding domain of fibronectin dramatically inhibits exosome-target cell interaction (59). The heparin-derived compound Roneparstat significantly inhibited interactions between exosomes and the target cells with a high safety profile in a phase 1 clinical trial (NCT01764880) (60). While the efficacy of Roneparstat still needs more investigation, interfering with fibronectin-heparan sulfate interactions to suppress exosome-mediated cross talk provides a novel insight to target myeloma tumor growth or progression.

However, despite the tumor-promoting and immunosuppressive effects of exosomes discussed previously, some studies showed that exosomes displaying high levels of heat shock protein 70 (HSP70) could boost NK cell responses (61). A study demonstrated that upon doxorubicin and melphalan treatment, MM cells significantly increased released exosomes that could stimulate interferon gamma (IFNγ) production, probably through mechanisms involving toll-like receptor (TLR) 2 and HSP70-dependent activation of the NF-κB pathway (62). Another study indicated that low doses of doxorubicin and melphalan could induce senescence to boost the expression of IL15/IL15RA complex on the surface of MM cells and their exosomes, promoting NK cell activation and proliferation (63). Therefore, suitable chemotherapeutic regimens may target and modulate exosomes to elicit anti-myeloma immune response. Tumor-derived exosomes as a source of tumor antigens for vaccines have also been explored. Xie et al. showed that membrane-bound HSP70-engineered myeloma cell-derived exosomes were able to induce DCs maturation and stimulate efficient CD4+ Th1, CD8+ CTL, and NK-mediated antitumor immunity. Membrane-bound HSP70 functioned both as an antigenic peptide chaperone and a danger signal that triggered DCs and therefore contributed significant adjuvant effects to exosome-based antitumor vaccine (64).

Besides providing novel therapeutic targets, the natural nanostructure and modifiable surface properties of exosomes make them a good candidate for drug delivery or immunomodulatory therapy. Knowing that cancer cells have selective sensitivity to TNF-related apoptosis-inducing ligand (TRAIL), Rivoltini et al. genetically modified cells to express TRAIL that could be subsequently embedded in secreted exosomes (65, 66). Those TRAIL-armed exosomes induced potent target cell apoptosis *in vitro* and controlled cancer progression when directly injected into tumor lesion. Though TRAIL exosomes had a preferential interaction with TRAIL-death receptor (DR) 5, *in vivo* study still showed increased areas of necrosis together with augmented levels of dead cells even in MM cells expressing other DR (66). Moreover, TRAIL exosomes can be easily produced in large amounts and stored before administration, making this a versatile, off-the-shelf therapeutic approach. Given their high stability in body fluids and natural delivery functions, TRAIL exosomes can also be loaded with drugs and genetic material and delivered to cancer cells through uptake process to elicit antitumor effects. Researchers are also exploring exosome-mimetic nanovesicles of similar sizes, morphologic features, and targeting abilities to replace exosomes in drug delivery (67, 68). With rapid technology advancement, engineering exosomes or their mimics to carry tumor-suppressive molecules or signals will, alone or in combination with other therapeutic approaches, contribute to innovative and effective MM treatment regimens.

## Conclusions and Future Perspective

Despite tremendous progress in the treatment of MM, this remains an incurable disease. It is well established that the BM microenvironment supports tumor in multiple aspects, while accumulating evidences demonstrate that exosomes plays a crucial role in the microenvironment network. Through selective transfer of mRNA, ncRNA, proteins, and lipids, exosomes are shown to mediate cell-to-cell communication and promote MM proliferation, drug resistance, immunosuppression, osteolysis, and angiogenesis. Recent studies are also investigating exosomes as potential biomarkers for MM diagnosis and predictive markers of drug response. In addition, the natural nanostructure of exosomes and their capacity to deliver molecules to target cells make them excellent drug carrier. Overall, exosomes offer the opportunity to both deepen our understanding of the molecular mechanism of MM pathogenesis and to provide a potential useful biomarker and therapeutic strategy in MM.

## Author Contributions

TC and GB conceived the project, wrote the initial draft, and finalized the manuscript. MM contributed to modifications of the initial draft. All authors contributed to the article and approved the submitted version.

## Conflict of Interest

The authors declare that the research was conducted in the absence of any commercial or financial relationships that could be construed as a potential conflict of interest.
